# The uncertain future

**DOI:** 10.1016/S1808-8694(15)30959-9

**Published:** 2015-10-19

*“When everything else is lost, the future is still there”*

(Bovee)

Today we face what may be the most uncertainty regarding the future of medicine and physicians in Brazil. Recent years have been branded by deep changes in all health-related fields. Starting by medical teaching, that so far counts on 156 Medical Schools. The 55 most recently created were inaugurated early this century, without going through a meticulous analysis of their true social need, but only to serve regional and political interests. Today, Brazil is at the top of the list of countries with the most medical schools, leaving behind China, with 150, India with 140 and the USA, with 125. In a country that in 1960 had 29 Medical Schools, this “outburst” of new medical teaching facilities is nothing but tragical. Thus, we annually see 13 thousand new physicians to graduate. In the year 2010, we will have about 60,000 new physicians. Moreover, about 2,500 Brazilians study medicine in Bolivia, 500 in Cuba, and just as many study in Argentina. If they do not manage to transfer themselves to our country during the course itself, they come back to Brazil after they graduate in an attempt to revalidate their diplomas and work here as physicians. Meanwhile, the USA is again, concerned with the high number of Medical Schools and specialists they have there, having seen signs of unemployment in the short run.

Public health care systems, limping for years, suffer from the sad and permanent lack of funds. Managerial incompetence and irregular use of funds are driving the system towards total bankruptcy, with loss and theft of millions of reais. Public hospitals and those maintained by Universities are perishing, without investments and funds they are but struggling to survive.

Since they have one single option, that is to care for SUS patients, based on ridicule and disparaging fees that do not even cover operational costs, face despair and pre-insolvency. The needy population is then left on their own, humiliated, uncertain of their “right to citizenship”. Service providers bemoan for having to play with the government, “they pretend to work and the government pretend to pay”.

It is within this unprecedented chaos that all sorts of health insurance plans thrive, increasingly betting on the failure of the public health system. Despite efforts from medical entities and consumers’ rights agencies, the regulation of health insurance plans serves only the claims of the big insurance firms and group medicine companies.

If this scenario does not change, the future may bring us a sad reality: physicians, in abounding numbers, becoming garden-variety employees, and the Brazilian medicine turning into a lucrative practice, a business like any other. Ethics, idealism, the sacerdotal spirit and physician-patient relationship are simple thrown away … “in the garbage bin”.

Dr. Antonio Celso Nunes Nassif is a physician. Associate Professor - UFPR. Former President of the Brazilian Medical Association.

acnnassif@netpar.com.br

